# Predictive value of metabolic score for insulin resistance and triglyceride glucose-BMI among patients with acute myocardial infarction in 1-year follow-up

**DOI:** 10.1097/MCA.0000000000001242

**Published:** 2023-05-23

**Authors:** Dominika Drwiła-Stec, Paweł Rostoff, Grzegorz Gajos, Jadwiga Nessler, Ewa Konduracka

**Affiliations:** a Department of Coronary Disease and Heart Failure at John Paul II Hospital; b Jagiellonian University Medical College, Kraków, Poland

**Keywords:** acute myocardial infarction, all-cause mortality, major adverse cardiovascular events, metabolic score for insulin resistance, triglyceride glucose-BMI

## Abstract

**Background:**

Recently two indicators – metabolic score for insulin resistance (METS-IR) and triglyceride glucose-BMI (TyG-BMI) have been proposed as surrogate markers of IR and potential cardiovascular risk factors. The aim of the study was to assess the predictive value of METS-IR and TyG-BMI concerning the incidence of major adverse cardiovascular events (MACE) and all-cause mortality in 1-year follow-up among patients admitted with acute myocardial infarction (AMI).

**Methods:**

Two thousand one hundred fifty-three patients with a median age of 68 years were enrolled in the study. Patients were divided into two groups according to the type of AMI.

**Results:**

MACE occurred in 7.9% of the patients in the ST-segment elevation myocardial infarction (STEMI) group and in 10.9% of the non-STEMI (NSTEMI) group. No significant difference in median MACE-IR and TyG-BMI between patients with and without incidence of MACE was found in both groups. None of the examined indices were predictors of MACE in the STEMI and NSTEMI groups. Moreover, both of them did not predict MACE in subgroups of patients classified according to the presence of diabetes. Finally, METS-IR and TyG-BMI were significant predictors of 1-year morality, however with low prognostic value and only in univariate regression analysis.

**Conclusion:**

METS-IR and TyG-BMI should not be used in predicting MACE among patients with AMI.

## Introduction

Cardiovascular disease remains a leading cause of death in highly developed countries therefore much effort is put into primary and secondary prevention of that disease. Even though the results of a recent study performed in Poland showed that management with basic lipid cardiovascular risk factors had improved over the last few years [1] the in-hospital and 1-year mortality of patients with acute myocardial infarction (AMI) is still high [2].

The results of many studies have shown that insulin resistance (IR) plays an important role in the development and progression of coronary artery disease (CAD) among patients with and without diabetes [3,4]. The gold standard for measuring IR is the Homeostatic Model Assessment-IR test. This, however, requires measurement of plasma insulin concentration which is not always possible in in-hospital and outpatient settings, therefore several surrogate noninsulin-based indices have been proposed as new tools for its measurement [5]. Because the prevalence of IR among CAD patients is high, these metabolic indices have been suggested as novel cardiometabolic risk factors; however, their predictive value among patients with diagnosed CAD remains unknown.

The primary aim of the current study was to assess the predictive value of the metabolic score for IR (METS-IR) and triglyceride glucose-BMI (TyG-BMI) regarding the incidence of major adverse cardiovascular events (MACE) in 1-year follow-up among patients with AMI. Secondarily, the value of these indices was evaluated separately in patients with and without diabetes. Finally, 1-year all-cause mortality was also evaluated.

## Methods

A retrospective, cohort study was conducted among 2300 consecutively recruited patients admitted to our department due to AMI.

Inclusion criteria were: diagnosis of ST-segment elevation myocardial infarction (STEMI) or non-STEMI (NSTEMI), haemodynamically relevant atherosclerosis present in the coronary angiography performed on admission and complete medical records.

Exclusion criteria were: AMI with nonobstructive CAD, cardiogenic shock on admission and incomplete medical history.

Urgent coronary angiography, followed by percutaneous coronary intervention (PCI) with stent implementation or coronary artery bypass grafting (CABG), was performed in all of the patients. Basic blood tests, echocardiography and anthropological measurements to determine patients’ BMI were performed. In addition, the Gensini score system was used to determine CAD severity [6].

The study protocol was completed in accordance with the Declaration of Helsinki and approved by the local Ethics Committee (The Jagiellonian University Medical College – KBET: 1072.6120.189.2020 to EK). Written informed consent was obtained from each study participant.

Data concerning MACE and all-cause mortality were obtained from anamnesis with patients or their families via telephone consultations, one year after hospital admission due to AMI.

### Laboratory tests

Glucose concentration and lipid profile were obtained from fasting blood samples collected within 24 h of admission. The direct enzymatic colorimetric method, using commercial in-vitro diagnostic devices (Cobas c, Roche, Switzerland) was used to determine the lipid profile, whereas the enzymatic hexokinase technique, using in-vitro diagnostic equipment (Cobas c) was used to measure glucose concentration. METS-IR and TyG-BMI were calculated manually according to the following formulas: METS-IR= [ln (2 × glucose + triglycerides) × BMI]/ln (high-density lipoprotein cholesterol); TyG-BMI = ln (triglycerides × glucose/2) × BMI. All of the lipid variables and glucose concentration were expressed in mg/dl whereas BMI was determined in kg/m^2^.

### Definitions

The fourth Universal Definition of Myocardial Infarction (2018) was used to define AMI [7]. MACE was a composite of unstable angina, AMI, in-stent restenosis, stroke or transient ischaemic attack and hospitalization due to heart failure. The use of antihypertensive drugs or a blood pressure of 140/80 mmHg or greater on at least two separate measurements was defined as hypertension. Diabetes diagnosed prior to current hospital admission or use of glucose-lowering drugs or insulin was defined as diabetes.

### Statistical analysis

The STATISTICA 13.3 software package (TIBCO Software Inc., Palo Alto, California, USA) was used to make all calculations. A two-sided *P* value < 0.05 was considered to be statistically significant. Categorical variables were expressed as numbers and percentages whereas continuous variables were shown as medians, using the first and third quartiles. The Shapiro–Wilk test was used to assess the normality of examined variables. The Mann–Whitney and Kruskal–Wallis tests were used for nonnormally distributed continuous variables and the chi-square test was used to compare categorical variables. Independent predictors of MACE and 1-year mortality were assessed using stepwise logistic regression analysis. The final multivariable model included variables that were significant univariate predictors.

## Results

For the initial analysis, 2300 patients admitted to our department were included. A total of five patients did not agree to participate in the study, 18 patients were excluded due to cardiogenic shock on admission and we did not obtain data concerning the incidence of MACE and mortality at 1-year follow-up from 124 individuals.

Finally, we analyzed data collected from 2153 patients (852 diagnosed with STEMI and 1301 with NSTEMI). The median age of study participants was 68 (60–77) years; 1462 (67.9%) were male. Most patients were overweight, with a median BMI of 27 (24.2–29.7) kg/m². For 1344 (62.4%) patients, current AMI was the first manifestation of CAD. The prevalence of hypertension and diabetes was 1838 (85.4%) and 807 (37.5%), respectively.

There were no statistically significant differences in the percentage of males, median Gensini score, TyG-BMI, METS-IR, HDL-cholesterol, triglycerides and left ventricular ejection fraction between patients with STEMI and NSTEMI. Patients with NSTEMI were older and had higher median BMI values. Moreover, almost half of them had been diagnosed with CAD prior to current admission. Finally, the incidence of hypertension, diabetes and MACE at 1-year follow-up was significantly higher in this group. Detailed characteristics of patients with STEMI and NSTEMI are shown in Table 1.

**Table 1:** Demographic and clinical characteristics of the study patients

Variables	Patients with STEMI *n* = 852	Patients with NSTEMI *n* = 1301	*P* value
Male sex, *n* (%)	590 (69.2)	872 (67)	0.2
Age, years^a^	65 (58–75)	70 (62–79)	<0.01
BMI, kg/m^2^^a^	26.7 (24.1–29.4)	27.2 (24.4–30)	0.02
CAD diagnosed prior to admission, *n* (%)	191 (22.4)	618 (47.5)	<0.01
LVEF	50 (40–55)	50 (35–55)	0.8
Hypertension, *n* (%)	662 (77.7)	1176 (90.4)	<0.01
Diabetes, *n* (%)	259 (30.4)	548 (42.1)	<0.01
Gensini score	52 (32–84)	56 (27–100)	0.4
eGFR, ml/min/1.73 m^2^^a^	57.9 (47.4–69.5)	55.5 (44.6–65.7)	<0.01
TyG-BMI	239.8 (213.3–272.5)	241.9 (213.9–275.2)	0.7
METS-IR	42.7 (37.1–48.7)	42.7 (37.2–49.4)	0.6
LDL-cholesterol, mmol/l^a^	2.8 (2.2–3.6)	2.6 (1.9–3.4)	<0.01
HDL-cholesterol, mmol/l^a^	1.15 (0.96–1.4)	1.16 (0.96–1.4)	0.7
Non-HDL-cholesterol, mmol/l^a^	3.2 (2.5–4.1)	3 (2.3–3.9)	<0.01
TC, mmol/l^a^	4.5 (3.8–5.3)	4.2 (3.5–5.1)	<0.01
Triglyceride, mmol/l^a^	1.3 (0.96–1.8)	1.3 (0.97–1.7)	0.3
Lipid-lowering therapy prior to admission, *n* (%)	694 (81.5)	1112 (85.5)	0.01
Incidence of MACE, *n* (%)	68 (7.9)	142 (10.9)	0.02
All-cause mortality, *n* (%)	92 (10.8)	174 (13.4)	0.07

CAD, coronary artery disease; eGFR, estimated glomerular filtration rate; LVEF, left ventricular ejection fraction; MACE, major adverse cardiovascular events; METS-IR, metabolic score for insulin resistance; NSTEMI, non-ST-segment elevation myocardial infarction; STEMI, ST-segment elevation myocardial infarction; TC, total cholesterol; TyG-BMI, triglyceride glucose-BMI.

a Data are shown as median (interquartile range) unless otherwise indicated. *P* < 0.05 was considered significant.

### Evaluation of ST-segment elevation myocardial infarction and non-ST-segment elevation myocardial infarction groups

During 1-year follow-up, MACE occurred in 68 (7.9%) patients with STEMI. There were 62 participants with a single incidence of MACE (29 cases of unstable angina, 15 cases of AMI, five cases of in-stent restenosis, eight hospitalizations due to heart failure and five cases of stroke or transient ischaemic attack). In addition, six individuals had two events (four cases of AMI and in-stent restenosis; one case of in-stent restenosis and hospitalization due to heart failure; one hospitalization due to heart failure and AMI).

There was no statistically significant difference in median METS-IR between patients with and without MACE at 1-year follow-up (*P* = 0.4). Furthermore, there was no difference between these groups for TyG-BMI (*P* = 0.5). In addition, the study found no statistically significant differences in the occurrence of diabetes, hypertension and the percentage of patients with previously diagnosed CAD among STEMI patients with and without MACE.

In the NSTEMI group, there were 142 (10.9%) cases of MACE. At the end of 1-year of follow-up, we reported 52 cases of AMI, 36 cases of unstable angina, four cases of in-stent restenosis, 15 hospitalizations for heart failure and seven cases of stroke or transient ischaemic attack. In addition, 27 patients had at least two incidents of MACE (17 cases of AMI with in-stent restenosis, seven cases of unstable angina and in-stent restenosis, one case of AMI and hospitalization for heart failure, one case of unstable angina and stroke or transient ischaemic attack). Finally, one patient who had an AMI with in-stent restenosis was also hospitalized due to heart failure.

Our study showed no statistically significant difference in median METS-IR and TyG-BMI among patients with and without MACE in the NSTEMI group (*P* = 0.9, *P* = 0.7 respectively). In contrast to the STEMI group, we observed significant differences concerning the median age of patients and the percentage of individuals with previously diagnosed CAD and diabetes. Detailed characteristics of the STEMI and NSTEMI groups are presented in Table 2.

**Table 2:** Detailed characteristics of the ST-segment elevation myocardial infarction and non-ST-segment elevation myocardial infarction groups

	Patients with STEMI			Patients with NSTEMI		
Variable	Incidence of MACE, *n* = 68	No MACE, *n* = 784	*P* value	Incidence of MACE, *n* = 142	No MACE, *n* = 1159	*P* value
Male sex, *n* (%)	45 (66.20)	545 (69.5)	0.6	98 (68)	774 (66.8)	0.6
Age, years^a^	66 (58.5–75.5)	65 (58–75)	0.5	73 (64–80)	70 (62–79)	0.02
BMI, kg/m^2^^a^	27.4 (24.5–30.70)	26.6 (24.1–29.4)	0.1	26.5 (24–30.1)	27.2 (24.4–30)	0.4
CAD diagnosed prior to admission, *n* (%)	16 (23.5)	175 (22.3)	0.8	91 (64.1)	527 (45.5)	<0.01
Lipid-lowering therapy prior to admission, *n* (%)	54 (79.4)	640 (81.6)	0.7	114 (80.3)	998 (86.1)	0.06
LVEF, %^a^	50 (35–55)	45 (30–50)	0.08	50 (40–55)	48 (35–60)	0.6
Hypertension, *n* (%)	53 (77.9)	609 (77.7)	0.9	131 (92.2)	1045 (90.2)	0.4
Diabetes, *n* (%)	21 (30.9)	238 (30.4)	0.9	79 (55.6)	469 (40.5)	<0.01
Gensini score	58 (34–84)	51 (32–84)	0.4	62 (27–113)	54 (27–96)	0.2
eGFR, ml/min/1.73 m^2^^a^	54.5 (47–65.5)	58.3 (47.5–70)	0.2	52.5 (40.3–64)	55.6 (45–65.9)	0.07
TyG-BMI	241.4 (211.8–282.3)	239.8 (213.3–272.3)	0.5	244 (212.3–272)	241.6 (213.8–275.4)	0.7
METS-IR	44.4 (36.5–51.2)	42.6 (37.1–48.7)	0.4	42.6 (36.9–50)	42.6 (37.1–49.3)	0.9

CAD, coronary artery disease; eGFR, estimated glomerular filtration rate; LVEF, left ventricular ejection fraction; MACE, major adverse cardiovascular events; METS-IR, metabolic score for insulin resistance; NSTEMI, non-ST-segment elevation myocardial infarction; STEMI, ST-segment elevation myocardial infarction; TC, total cholesterol; TyG-BMI, triglyceride glucose-BMI.

a Data are shown as median (interquartile range) unless otherwise indicated. *P* < 0.05 was considered significant.

### Predictors of major adverse cardiovascular events

In the STEMI group, no statistically significant predictor for MACE was found. Both METS-IR and TyG-BMI were insignificant in the univariate analysis with *P* = 0.3 and *P* = 0.2, respectively.

Similarly, in the NSTEMI group, neither of the evaluated indices were significant predictors of MACE (*P* = 0.79 for METS-IR, *P* = 0.77 for TyG-BMI). In univariate analysis, age, diabetes and CAD diagnosed prior to admission were found to be significant indicators of these events [odds ratio (OR) = 1.02 (95% confidence interval [CI]: 1–1.03, *P* = 0.03) for age, OR = 1.8 (95% CI: 1.3–2.6, *P* < 0.01) for diabetes, OR = 2.1 (95% CI: 1.5–3.07, *P* < 0.01) for CAD diagnosed prior to admission] Furthermore, diabetes and CAD diagnosed before admission remained independent predictors of MACE in the multivariable model [OR = 1.7 (95% CI: 1.2–2.4, *P* < 0.01), OR = 2.1 (95% CI: 1.4–3, *P* < 0.01) respectively].

### Predictors of major adverse cardiovascular events in subgroups

To deepen the analysis, the STEMI and NSTEMI groups were divided according to the presence of diabetes.

Among patients with STEMI and diabetes, there was no difference in median METS-IR and TyG-BMI between patients with and without MACE (*P* = 0.1, *P* = 0.3, respectively). Similar results were obtained for STEMI patients without diabetes (*P* = 0.9, *P* = 0.9, respectively).

Neither indices were a predictor of MACE among patients with or without diabetes in the STEMI group (*P* = 0.13, *P* = 0.9 for METS-IR, *P* = 0.16, *P* = 0.7 for TyG-BMI, respectively). Only BMI showed significance as an independent predictor of MACE among patients with diabetes in the STEMI group [OR = 1.3 (95% CI: 1.05–1.6, *P* = 0.02)], whereas no predictor was found in patients without diabetes.

The results obtained in the NSTEMI group correspond with findings from the STEMI group. There were no significant differences in median METS-IR and TyG-BMI among patients with diabetes (*P* = 0.8, *P* = 0.4, respectively) and without diabetes (*P* = 0.4, *P* = 0.3, respectively).

None of the evaluated indices appeared to be predictors of MACE among both patients with and without diabetes (*P* = 0.6, *P* = 0.5 for METS-IR, *P* = 0.3, *P* = 0.4 for Tyg-BMI). Among NSTEMI patients with diabetes, only CAD diagnosed prior to admission and no lipid-lowering therapy before admission were indicators of MACE. Moreover, both parameters remained independent predictors in the multivariable model [OR = 1.8 (95% CI: 1.1–2.9, *P* = 0.02) for CAD diagnosed prior to admission, OR = 2.1 (95% CI: 1.2–3.8, *P* = 0.01) for the absence of lipid-lowering therapy prior to admission].

In addition, among NSTEMI patients without diabetes, only CAD diagnosed prior to admission was a significant predictor of MACE; moreover, it remained an independent predictor in the multivariable model [OR = 2.5 (95% CI: 1.5–4.3, *P* < 0.01)].

### Predictors of 1-year mortality

The 1-year mortality rate was 12.3% (*n* = 266). Table 3 shows statistically significant predictors of 1-year all-cause mortality. Importantly, both METS-IR and TyG-BMI were predictors of all-cause mortality in univariate analysis, but with borderline significance; however, only age, BMI and estimated glomerular filtration rate (eGFR) remained independent predictors in the multivariable model [OR = 1.08 (95% CI: 1.05–1.1, *P* < 0.01) for age; OR = 0.8 (95% CI: 0.73–0.95, *P* < 0.01) for BMI; OR = 0.98 (95% CI: 0.97–1, *P* < 0.01) for eGFR].

**Table 3:** Predictors of all-cause mortality in 1-year follow-up (univariate regression analysis)

Predictors of all-cause mortality in 1-year follow-up	OR	95% CI	*P* value
Age	1.08	1.06–1.09	<0.01
BMI	0.9	0.87–0.94	<0.01
Gensini score	1.0	1–1.02	<0.01
eGFR	0.97	0.96–0.98	<0.01
No lipid-lowering therapy prior to admission	1.7	1.2–2.3	<0.01
METS-IR	0.97	0.95–0.98	<0.01
TyG-BMI	0.99	0.98–0.99	<0.01

CI, confidence interval; eGFR, estimated glomerular filtration rate; METS-IR, metabolic score for insulin resistance; OR, odds ratio; TyG-BMI, triglyceride glucose-BMI.

Supplementary Table 1, Supplemental digital content 1, http://links.lww.com/MCA/A562 shows medical treatment at discharge.

## Discussion

Due to the high prevalence of IR among patients with CAD, cardiometabolic biomarkers have received worldwide attention from researchers in recent years. It has been suggested that cardiometabolic indices might be useful and also sensitive cardiovascular risk factors to support classical, commonly used concentrations of key lipoproteins. In our previous study, we assessed the predictive value of one such index, the triglyceride-glucose index (TyG index) among nondiabetic patients with AMI [8]; however, that study showed that this index was not a predictor of MACE and 1-year mortality.

In the current study, we focused on the predictive value of two other cardiometabolic indices, METS-IR and TyG-BMI.

The METS-IR was originally introduced by Bello-Chavolla *et al*. [9] as a tool to evaluate insulin sensitivity and detect IR. The authors concluded that METS-IR shows good diagnostic efficacy and, in addition, the use of fasting glucose, triglycerides and HDL-C along with BMI make this marker simple, low-cost and easily accessible to primary care physicians.

We found no study regarding the predictive value of METS-IR among patients with AMI; moreover, at the time of writing this article, only one paper on METS-IR as a cardiovascular risk factor was available.

Yoon *et al*. [10] assessed whether METS-IR is a predictor of incidental ischaemic heart disease in a large cohort of nondiabetic patients. The study showed that the hazard ratio for developing ischaemic heart disease during follow-up increased with higher quartiles of METS-IR and was highest in the top quartile of METS-IR (values ≥38.0). Furthermore, the cut-off value of METS-IR to predict ischaemic heart disease of 31.1 was considered optimal. Although the study of Yoon *et al*. did not assess the prognostic value of METS-IR among patients with ischaemic heart disease, we can compare some findings. In our study, the median METS-IR was 42.7 in the STEMI and NSTEMI group, with values at the first quartiles much higher than 31.1, therefore the suggestion that such value may predict CAD is consistent with our results.

The second cardiometabolic biomarker assessed in our study, the TyG-BMI, was first introduced by Er *et al* [11]. The authors demonstrated the superiority of TyG-BMI in identifying individuals with IR over other surrogate markers (including the TyG index) among nondiabetic patients. Another study on this subject reported that because obesity is strongly associated with IR, the combination of TyG and anthropological parameters including BMI might be better in detecting individuals with IR [12]. In addition, a large cross-sectional study, performed in the USA [13], revealed optimal cut-off values for TyG-BMI to detect IR. These values were 135.5 for both men and women. In our study population, these values were much higher, therefore, it can be assumed that IR occurred at a high frequency in our study.

To the best of our knowledge, this study is the first to assess the predictive value of the TyG-BMI in AMI patients, with CAD confirmed in coronary angiography.

A study by Cho *et al*. [14] assessed the predictive value of TyG-related markers on cardiovascular risk by evaluating the progression of coronary artery calcification in noncontrast multidetector computed tomography. In this study, there was a significant difference in mean TyG-BMI between patients with and without progression of coronary calcification (224.2 ± 29.0 vs 216.2 ± 34.6). The OR for the progression of coronary artery calcification was significant only for the highest quartile of TyG-BMI; however, the authors did not show the range of TyG-BMI values in this group, therefore a comparison with our study is not possible. Furthermore, patients with a history of CAD, statin use, and PCI or CABG were excluded from the study of Cho *et al*. The authors suggest that another TyG-related marker, TyG-waist circumference, is superior and only this parameter has been recommended for cardiovascular risk estimation in everyday clinical practice. In our study, TyG-BMI did not predict MACE in any group. Moreover, as a predictor of all-cause mortality, it showed borderline significance only in univariate analysis. Therefore, we agree with the conclusions of Cho *et al*. regarding the lack of recommendations for the everyday use of TyG-BMI.

Huang *et al*. [15] performed a study assessing the predictive value of TyG-BMI on the 10-year risk of atherosclerotic cardiovascular disease (ASCVD) in comparison with other cardiometabolic markers. In that study, ASCVD was defined as the risk of nonfatal AMI, death from coronary heart disease and nonfatal or fatal stroke. Two groups of patients, low and high risk of ASCVD, were identified. The median TyG-BMI values were higher in the high ASCVD risk group in both men and women. In addition, TyG-BMI was a predictor of high ASCVD risk unadjusted and also after adjustment for other parameters. In the Huang *et al*. study, the ASCVD risk was assessed using the Pool Cohort Equations, which are based on several cardiometabolic variables: furthermore, there was no follow-up, thus TyG-BMI was actually a predictor of calculated risk. We do not know whether the results of that study would have been different if there had been a follow-up.

## Study limitations

Although our study is novel in terms of determining the predictive and prognostic values of METS-IR and TyG-BMI in patients with AMI, it has several limitations. First, this was a single-centre study; therefore, a multiregional and multiethnic study is needed to determine the prognostic value of the studied parameters in the future. Second, potential changes in METS-IR and TyG-BMI values during follow-up were not evaluated. Third, there is no information on changes in medical treatment during follow-up and patients' compliance with prescribed therapy. Finally, the follow-up period was relatively short.

### Conclusion

Because our study is the first to assess the predictive value of METS-IR and TyG-BMI in patients with AMI, further studies should be performed to unambiguously determine the clinical importance of these parameters. Currently, however, there is no evidence that both METS-IR and TyG-BMI can be used in risk stratification in patients with AMI. Furthermore, the borderline significance in predicting 1-year mortality with no relevance in multivariable models also reflects the questionable clinical value of these parameters. Risk stratification of AMI patients should focus on basic risk factors and well known and widely evaluated scales.

## Acknowledgements

### Conflicts of interest

There are no conflicts of interest.

## Supplementary Material


