# Supplementary material for: Predictive value of metabolic score for insulin resistance and triglyceride glucose-BMI among patients with acute myocardial infarction in 1-year follow-up
Source: Coron Artery Dis. 2023 May 23;34(5):314–9. doi: 10.1097/MCA.0000000000001242 (PMC10758348; doi:10.1097/MCA.0000000000001242)
Supplement: Supplementary file 1 [file cad-34-314-s001.pdf]

Supplementary Table 1. Medical treatment at discharge.

Medical treatment at discharge	Number of patients (%)
Statins, n (%)	2142 (99.5)
Ezetimibe, n (%)	375 (17.4)
Fibrates, n (%)	3 (0.13)
ACEI, n (%)	1115 (51.8)
ARB, n (%)	680 (31.6)
B-adrenolitics, n (%)	1901 (88.3)
Calcium blockers, n (%)	1238 (57.5)
Diuretics, n (%)	961 (44.6)
ASA, n (%)	2151 (99.9)
Clopidogrel, n (%)	1927 (89.5)
Prasugrel/Ticagrelor, n (%)	241 (11.2)
Metformin, n (%)	722 (33.5)
Sulfonylurea derivatives, n (%)	50 (2.3)
SGLT-2 inhibitors, n (%)	103 (4.8)
DPP-4 inhibitors, n (%)	2 (0.09)
Insulin, n (%)	78 (3.6)

Abbreviations: ACEI, angiotensin-converting enzyme inhibitors; ARB, Angiotensin receptor blockers; ASA, aspirin; SGLT, Sodium-glucose Cotransporter-2; DPP, Dipeptidyl peptidase.
